# Grooming Device Effects on Behaviour and Welfare of Japanese Black Fattening Cattle

**DOI:** 10.3390/ani9040186

**Published:** 2019-04-23

**Authors:** Shigeru Ninomiya

**Affiliations:** 1 Faculty of Applied Biological Sciences, Gifu University, 1-1 Yanagido, Gifu 501-1193, Japan; nino38@gifu-u.ac.jp; Tel.: +81-58-293-2894; 2 Donated Fund Laboratory of Animal Welfare (Ishii), Graduate School of Agricultural Science, Tohoku University, 232-3, Yomogita, Naruko-Onsen, Ohsaki, Miyagi 989-6711, Japan

**Keywords:** animal welfare, environmental enrichment, grooming device, fattening cattle

## Abstract

**Simple Summary:**

Fattening cattle housed at a private farm in Japan were provided with a grooming device (a brush) during the late fattening stage. Behavioural observation revealed that the environmental enrichment stimulated the animals’ self-grooming and enabled them to scratch more body parts. Viscera disease was not detected in the enrichment animals when they were slaughtered. Environmental enrichment for fattening cattle can be used to satisfy their motivation to perform self-grooming and to improve their welfare.

**Abstract:**

In livestock farming, a stark or barren environment compromises animal welfare. Environmental enrichment has been used to address the issue. For this study, after fattening cattle were provided with a grooming device (a brush), its effect on animal self-grooming and welfare were investigated. For Research trial 1 and 2, respectively, 28 and 11 Japanese Black steers were observed. Three or four of the animals were group-housed in a pen. For Trial 1, half of the animals were provided with a brush. The animals’ behaviour, carcass weight, and Viscera disease were recorded. Enrichment animals (E) performed self-grooming and scratching of the animals’ body on the brush and pen structures more than control animals (C) did (mean time budgets, 3.34% (SD = 2.48) in E and 0.89% (SD = 0.81) in C, GLMM, z value = 8.28, *p* < 0.001). The number of animals in which viscera disease was detected after slaughter was lower in E than in C (E = 0, C = 4, a Fisher’s exact probability test, *p* = 0.03). In Trial 2, brush use behaviour was observed continuously for 72 h. The observation revealed that the animals scratched various body parts on the brush. Results show that providing a brush as environmental enrichment improves welfare by satisfying the motivation of fattening cattle to perform self-grooming.

## 1. Introduction

For livestock farming, intensive housing systems have been developed mainly from an economic perspective. However, the systems have been reconsidered from the viewpoint of farm animal welfare. Some intensive housing systems (e.g., a battery cage for laying hens, stalls for sows) have been replaced to a remarkable degree in the past decade by systems that provide animals with freedom to express normal behaviour [1]. Behavioural restrictions in an artificial environment are harmful for animal welfare [2] because they might induce animals’ frustration and maladaptation to their environment, which would worsen their physical and psychological health [3]. To reduce the risk of behavioural restrictions, adequate animal management for decreasing the sources of stressors or for providing environmental enrichment is suggested [3].

Housing systems used on Japanese fattening cattle farms are also intensive. Maintaining productive efficiency per unit of area and labour is important for economic rationality. For instance, stock density and the frequency of bedding and muck management tend to be decided mainly according to economic constraints. Because of this management, the bedding conditions can make the animal’s body dirty and might motivate them to perform self-grooming behaviour. However, animals do not always express self-grooming effectively in an artificial environment. This inadequacy of grooming consequently induces their frustration and compromises their welfare. For improving cattle welfare, keeping the bedding clean and increasing the frequency of bedding and muck management would be effective, but it is less possible to implement these operations because this will be costly for cattle farming based on labour efficiency and economic constraints. However, environmental enrichment would also resolve the related difficulties. Reportedly, beef cattle [4,5,6,7] and dairy cattle [8,9] use grooming devices well.

Therefore, as environmental enrichment in this study, a brush for self-grooming was installed at a private farm of fattening cattle in Japan. Cattle’s self-grooming in a farming bedding condition was managed and evaluated from an economic perspective. This study was conducted to investigate enrichment effects on self-grooming of fattening beef cattle and their welfare comparing with control conditions prevailing on the same farm.

## 2. Materials and Methods 

This study was conducted according to Standards relating to the Care and Keeping and Reducing Pain of Laboratory Animals in Japan and Regulations for Animal Experiments and Related Activities at Tohoku University.

### 2.1. Animals and Management

#### 2.1.1. Trial 1

At one private farm in Miyagi prefecture, Japan, 28 Japanese Black steers were examined. The animals were divided into two groups in terms of purchase time. Animals of Group A and B were approximately 29 and 26 months old at the start of Trial 1 and were in the late fattening stage. They had been purchased from other private farms at approximately 10 months of age.

**Figure 1:**
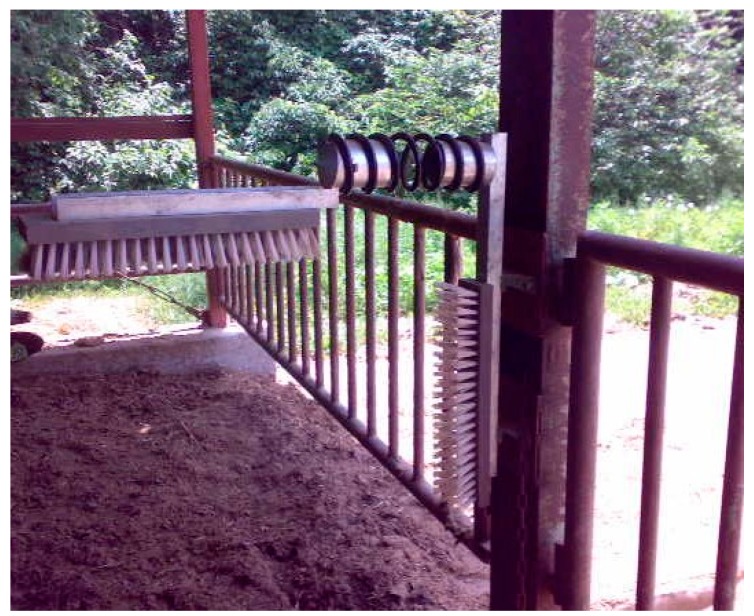
Grooming device (brush) used in this study.

Groups A and B included four subgroups; each subgroup comprised three or four animals housed in a 3.6 m × 7.2 m pen in the same housing of the farm. Two subgroups of groups A and B were housed in neighbouring pens. One of the two subgroups was provided with a brush; the other was a control subgroup. One brush measuring 50 cm long × 10 cm wide was placed together with a second brush in an inverted L-shaped pattern (Albert Kerbl, Buchbach, Germany). One brush was fixed horizontally about 110 cm from the floor. The other brush was attached to the post of the pen (Figure 1). The animals were slaughtered at around 32 months old. The mean research periods of Group A and B were about 120 and 204 days.

At the farm, concentrate feed was given to steers according to their fattening stage. The required amount of feed was provided in the morning. The proper quantity of rice straw (in general about 1kg per day for one animal) was also provided as forage three times each day. They were given free access to water. The bedding was removed about every 10 days. New sawdust bedding was added to the pens. The floor was solid concrete. The animals were reared on the farm according to the Act on Welfare and Management of Animals and Standards related to the Care and Keeping of Industrial Animals in Japan.

#### 2.1.2. Trial 2

Another 11 Japanese Black steers were used at the same farm. In trial 2, there were three groups and each group included three or four animals. The mean ages of the animals were 26–32 months. The management methods and housing in Trial 2 were identical to those of Trial 1. The same grooming devices were attached into each pen. The attachment method was identical to Trial 1.

### 2.2. Data Sampling

#### 2.2.1. Trial 1

Behavioural observation was conducted 32 days after providing a brush in the enrichment pens. Two observers conducted behavioural observations from 9:00 to 16:00. One observed Group A; the other observed Group B. They used the sampling methods described below. They conducted behavioural observations of the neighbouring two pens of the groups for 5 min; then they observed the other neighbouring two pens of the groups for 5 min. They repeated these observations. Scratching the animal’s own body on the brush or pen structures and licking of the own body were recorded every 30 s. Also, the body parts (head, neck, back, tail regions) were recorded. Eating, ruminating, standing resting, and lying resting were recorded every 10 min using time sampling. Table 1 showed the ethogram of the time sampling.

Scratching of the animal’s own body on the brush in Group A and B was again recorded 84 days and 178 days after providing the brush.

The time for slaughtering steers was decided by the farm for its market operations. The slaughter procedure was conducted under the Slaughterhouse Act, the Act on Welfare and Management of Animals and Standards related to the Methods of Destruction of Animals in Japan. During the procedure, a public institution checked each animal. For this study, the dressed carcass weights and prevalence of viscera diseases were referred to from reports made by the slaughterhouse. 

#### 2.2.2. Trial 2

More than 6 months after the enrichment, the animals’ brush use was recorded for three consecutive days using a CCD camera; 72 h of video were recorded on the video recorder. During the night, infrared light was used in the dark condition to observe the animals’ brush use. From the videos, brush use was recorded using continuous recording. Body parts that the animals scratched on the brush were recorded in detail by comparison with Trial 1 and these were head, neck, foreleg back, rib, hip cross, hindleg and tail. As brush use behaviour, ‘lick the brush’ and ‘hit the brush with its own horn’ were also recorded. 

### 2.3. Statistical Analysis

#### 2.3.1. Trial 1

During Trial 1, one steer from one control pen of Group B was removed to another housing because of agonistic behaviour from other steers of the pen. The animals were not investigated after the 32 days of observation. During the 32 days of observation, feeding at 16:00 started 20 min earlier than the usual time. During the 84 days observation of Group A, feeding at 9:00 was delayed to be 10 min later than the usual time. The observation periods during the 32 days and 84 days were for 9:00–15:40 and for 9:10–16:00.

The time budget of each behaviour being sampled using a time sampling was calculated as a proportion of the recorded number for the total observation period. For grooming behaviours, data of each body part were also calculated. 

For statistical analysis, the total time budgets of scratching of the animal’s body on the brush and pen structures and licking of the body by the enrichment animals were compared with those of control animals using a generalized linear mixed model. In the model, the treatment (enrichment or control) was used as a fixed factor. Two neighbouring pens were set as a block; blocks were used as a random factor. The data of scratching of the animal’s body on the brush for 32 days after the enrichment were compared with such data 84 days or 178 days after the enrichment in Group A or Group B using a generalized linear mixed model. In the model, the sampling day was used as a fixed factor. Blocks (each neighbouring two pens) were used as a random factor. All response variables were assumed to follow the Poisson distribution. The generalized linear mixed model was conducted using an R package (The R Foundation for Statistical Computing, Vienna, Austria).

For the enrichment and control, the dressed carcass weights were compared using a general linear model after the data were assessed to ascertain whether the assumptions of parametric testing had been met. The general linear model was applied using statistical software (Minitab ver. 14; Minitab Inc., State College, PA, USA). In the model, the treatment (enrichment or control) was used as a fixed factor. Two neighbouring pens were set as a block. Blocks were used as a random factor.

In public reports of viscera diseases, four animals were checked for liver diseases and one of them was also checked for an intestinal disease. The public report of viscera diseases of one steer from one control pen of Group B was not obtained. Using Fisher’s exact probability test, the occurrence probabilities of viscera disease in enrichment and control animals were compared.

#### 3.3.2. Trial 2

Because of the difficulty of individual identification from the videos, brush use data in Trial 2 was recorded without individual identification and figures in each group were divided by the number of animals of each group. The mean times and total durations of scratching each cattle’s body region on the brush per hour were calculated, and the total amount of brush use was also calculated. 

## 3. Results

### 3.1. Trial 1

The mean time budget of brush use 32 days after providing the brush was 2.80% (SEM = 0.63). Although scratching of their own back and tail on pen structures was seldom recorded in control animals, enrichment animals scratched their back and tail on the brush (Table 2). The total mean time budgets of self-grooming and scratching animals’ body on the brush and pen structures in enrichment animals (E) was 3.34% (SEM = 0.66 (%)) and those in control animals (C) was 0.89% (SEM = 0.22). A statistically significant difference was found between E and C (*z*-value = 8.28, *p* < 0.001). No statistically significant difference was found for other behavioural data between E and C (Table 3).

The mean time budget of brush use 84 days after providing the brush in Group A was 1.8% (SEM = 0.6). The data were lower than those recorded 32 days after providing the brush (*z*-value = 3.17, *p* < 0.01). The mean time budget of brush use 178 days after providing the brush in Group B was 2.4% (SEM = 0.4); no statistically significant difference was found between the data obtained 32 days and 178 days after providing the brush in Group B (*z*-value = 0.12, *p* = 0.90).

No statistically significant difference was found in the mean weight of the dressed carcass between E (420 kg (SEM = 9 kg)) and C (434 kg (SEM = 9 kg)) (*F*-value = 2.06, *p* = 0.17). Four animals in C were checked for liver diseases and one of them was also checked for an intestinal disease and therefore the number of animals for which viscera were checked because of disease was 0 in E and 4 in C. A statistically significant difference was found between the numbers (*p* = 0.03). 

### 3.2. Trial 2

The mean times (per hour, ± SEM) and total durations (seconds per hour, ± SEM) of the total amount of brush use were 1.23 ± 0.52 and 22.70 ± 11.04. Table 4 shows the mean frequency and total durations of scratching each cattle’s body region on the brush. The animals often scratched their head, neck, back, and ribs.

## 4. Discussion

From the two research activities used for this study, we infer that providing the brush increased the time budgets of self-grooming behaviour in the fattening steers housed in a private farm and enabled them to scratch more body parts on it. These results support those of earlier studies. Results showed that feedlot cattle use self-grooming devices more than scent devices [5]. A grooming device increases the total time spent by dairy cattle scratching their body parts [8]. Cows are highly motivated to access a grooming device [10].

In Group A, brush use was less frequent 84 days after providing the brush compared to data of 32 days, but no decrease was found at 178 days in Group B. Results of an earlier study suggest that use of a grooming device by cattle was a low-resilience activity that decreases under a range of conditions [9]. The possibility exists that, at around 84 days after providing the brush, the housing conditions of this study temporarily decreased the cattle’s motivation to use the brush. However, in the data of 178 days in Group B and the data of Trial 2 which were obtained at least 6 months after providing the brush, the animals used the brush continuously, which indicates that the grooming device in this study stimulated the animal’s self-grooming and insured expression of their self-grooming for more than six months.

As for the effect of providing the brush and insuring expression of the animals’ self-grooming, viscera disease was not detected in enrichment animals in this study. Moreover, a statistically significant difference was found in the prevalence of the disease between enrichment and control animals. No physiological explanation that the enrichment decreased viscera disease could be inferred from the results of this study because physiological data were not sampled, but it is possible that cattle welfare improvement from insuring expression of the animals’ self-grooming adequately might beneficially affect viscera health in cattle. However, from a post hoc power analysis using a G*Power software [11], the power level of the statistical analysis was 0.607 and it is thought that a further investigation for reproducibility or a high probability of the result would be needed.

In an artificial environment, animals do not always express normal behaviour adequately. This fact would lead to their frustration and compromise their welfare. Adequate animal welfare management (e.g. decreasing stressor, environmental enrichment) will resolve some problems of impoverished or barren environment [3]. Although this study did not show whether brush use in the animals was motivated by external factors (e.g., bedding management in the fattening farm) or behavioural needs [12] for self-grooming of the animals, providing the brush is expected to enable cattle to perform self-grooming adequately according to their motivation and is expected to improve their welfare.

## 5. Conclusions

Animals at a fattening farm investigated in this study were motivated to perform self-grooming. Providing a brush as environmental enrichment is expected to improve their welfare by satisfying their motivation to perform self-grooming.

## Figures and Tables

**Table 1:** Ethogram of the 10 min time sampling in Trial 1.

Behaviour	Definition
eating	ingest feed
ruminating	perform rumination
standing resting	be motionless in a standing position
lysing resting	be motionless in a lying position

**Table 2:** Mean time budgets (% ± SEM) of scratching cattle’s body on brush or pen structures in enrichment and control animals in Trial 1.

Behaviour	Enrichment	Pen Structure	Control
	Brush		Pen Structure
total	2.80 ± 0.63	0.54 ± 0.17	0.86 ± 0.22
head	0.70 ± 0.25	0.32 ± 0.10	0.41 ± 0.16
neck	0.41 ± 0.13	0.09 ± 0.07	0.39 ± 0.14
back	1.04 ± 0.32	0.11 ± 0.08	0.04 ± 0.02
tail	0.66 ± 0.48	0.02 ± 0.02	0.02 ± 0.02

Total means the total value of four body regions in brush or pen structure data of each group.

**Table 3:** Mean time budgets (% ± SEM) of each behavioural category in enrichments and control animals in Trial 1.

Behaviour	Enrichment	Control	*p*-Value
eating	18.6 ± 1.4	15.4 ± 1.3	0.19
ruminating	15.4 ± 1.3	13.6 ± 1.3	0.43
standing resting	25.5 ± 2.7	26.3 ± 2.0	0.81
lying resting	35.0 ± 2.3	38.9 ± 2.4	0.28

**Table 4:** Mean times (per hour ± SEM) and total durations (seconds per hour ± SEM) of the total amount of brush use and scratching each cattle’s body region on the brush in Trial 2.

Behaviour	Times	Total Durations
total	1.23 ± 0.52	22.70 ± 11.04
head	0.43 ± 0.13	6.17 ± 2.14
neck	0.27 ± 0.04	4.75 ± 1.09
foreleg	0.00 ± 0.00	0.02 ± 0.01
back	0.14 ± 0.13	3.20 ± 2.98
rib	0.11 ± 0.11	3.06 ± 3.06
hip cross	0.09 ± 0.07	1.70 ± 1.35
hindleg	0.04 ± 0.03	1.26 ± 0.75
tail	0.05 ± 0.05	1.04 ± 1.01
lick the brush	0.10 ± 0.03	1.43 ± 0.53
hit the brush with its own horn	0.00 ± 0.00	0.06 ± 0.06

Total means the total value of all body regions.
